# Quantitative analysis of nanoparticle internalization in mammalian cells by high resolution X-ray microscopy

**DOI:** 10.1186/1477-3155-9-14

**Published:** 2011-04-10

**Authors:** Hsiang-Hsin Chen, Chia-Chi Chien, Cyril Petibois, Cheng-Liang Wang, Yong S Chu, Sheng-Feng Lai, Tzu-En Hua, Yi-Yun Chen, Xiaoqing Cai, Ivan M Kempson, Yeukuang Hwu, Giorgio Margaritondo

**Affiliations:** 1 Institute of Physics, Academia Sinica, Nankang, Taipei 115, Taiwan; 2 Department of Engineering and System Science, National Tsing Hua University, Hsinchu 300, Taiwan; 3 Université de Bordeaux, CNRS UMR 5248, B8 Avenue des faculties, 33402 Talence-Cedex, France; 4 National Synchrotron Light Source II, Brookhaven National Laboratory, Upton, NY 11973, USA; 5 Institute of Optoelectronic Sciences, National Taiwan Ocean University, Keelung 202, Taiwan; 6 Ecole Polytechnique Fédérale de Lausanne, CH-1015 Lausanne, Switzerland

## Abstract

**Background:**

Quantitative analysis of nanoparticle uptake at the cellular level is critical to nanomedicine procedures. In particular, it is required for a realistic evaluation of their effects. Unfortunately, quantitative measurements of nanoparticle uptake still pose a formidable technical challenge. We present here a method to tackle this problem and analyze the number of metal nanoparticles present in different types of cells. The method relies on high-lateral-resolution (better than 30 nm) transmission x-ray microimages with both absorption contrast and phase contrast -- including two-dimensional (2D) projection images and three-dimensional (3D) tomographic reconstructions that directly show the nanoparticles.

**Results:**

Practical tests were successfully conducted on bare and polyethylene glycol (PEG) coated gold nanoparticles obtained by x-ray irradiation. Using two different cell lines, EMT and HeLa, we obtained the number of nanoparticle clusters uptaken by each cell and the cluster size. Furthermore, the analysis revealed interesting differences between 2D and 3D cultured cells as well as between 2D and 3D data for the same 3D specimen.

**Conclusions:**

We demonstrated the feasibility and effectiveness of our method, proving that it is accurate enough to measure the nanoparticle uptake differences between cells as well as the sizes of the formed nanoparticle clusters. The differences between 2D and 3D cultures and 2D and 3D images stress the importance of the 3D analysis which is made possible by our approach.

## Background

Quantitative analysis is an important but still largely unexplored issue in the study of nanomedicine procedures, in particular at the cellular and subcellular levels. Many phenomena were discovered by which nanoparticles enhance the cancer cell mortality or facilitate the action of other cell-killing factors [1-4]. However, the potential modulation of these phenomena for procedures such as radiotherapy [5-9] or drug delivery [7,10-13] requires clarifying a number of issues, many of them quantitative.

Such issues are not simple since each cell line interacts differently with nanoparticles [14-16]. Furthermore, the specific chemistry and morphology of each type of nanoparticles influence the interaction mechanisms leading to nanoparticle uptake [17-23]. Quantitative features are specifically important since they can affect internalization processes (endocytosis, pinocytosis, free membrane trafficking, etc.) [24-27], the optimization of nanomedicine procedures (in particular the maximum nanoparticle uptake by each cell line [28-30]) and the conditions to avoid toxicity.

An effective quantitative analysis should include not only average properties but also the statistical distributions for the level of uptake and for the size of the clusters formed by aggregated nanoparticles. Furthermore, it would be preferable to identify the location of the internalized nanoparticles and clusters with respect to the different organelles in cells for their different functions.

The procedure presented here meets these requirements and stems from an extensive previous work to develop suitable instruments and methods. In recent years, we introduced a series of imaging approaches for biosystems based on the high brightness and coherence of x-ray synchrotron sources [31-37]. Such methods reached sufficient spatial resolution for subcellular analysis [37], thus enabling us to harvest valuable and reliable quantitative information.

The results presented below show that the extraction of detailed quantitative data on nanoparticle cellular uptake is entirely feasible. Although so far validated for the specific case of gold nanoparticles (AuNPs) on two cell lines, the method can have much broader applications - for example, to all nanoparticles containing high-Z elements. The approach is non-destructive and reaches high spatial resolution.

The procedure started with the acquisition of transmission hard-x-ray micrographs with an instrument that can reach a 30-nm spatial resolution [38,39]. We collected either individual projection micrographs or sets of projection images at different angles for tomographic 3D reconstruction. The high penetration of our hard-x-rays (8 keV photon energy) made it possible to work with 3D samples, i.e., cell cultures in gel.

Large cell collections could be simultaneously imaged as required for quantitative analysis. Staining with heavy metals (uranium or osmium acetate) was used in specific cases to reveal specific intracellular (organelle) details. Zernike phase contrast was also exploited for visualizing nanoparticle clusters smaller than ~100 nm.

From the microimages, we extracted quantitative data on the number and size of uptaken nanoparticle clusters and information on the cluster positions in the cells. The procedure was first tested on bare (uncoated) AuNPs with average size ~15 nm prepared by a recently developed method [40-43]. This is based on x-ray irradiation of precursor solutions and produces nanoparticle colloids with high density and excellent stability. Although the sizes of these nanoparticles are smaller than the currently achieved resolution of X-ray microscopy, the aggregation of the nanoparticles after internalization by cells forms clusters of size large enough to be imaged and quantitatively analyzed.

The tests were then extended to AuNPs coated with polyetheleneglycol (PEG), prepared with a similar irradiation method [40]. We tested both types of nanoparticles on two different cancer cell lines, EMT-6 and HeLa cell, detecting the significant quantitative differences discussed below.

One interesting issue analyzed in our tests was the quantitative relation between the nanoparticle uptake and the cell survival. The image analysis results were cross-checked with those of cell viability bioassays. The corresponding conclusions are interesting on their own considering the present open issues on the cellular effects of AuNPs.

Specifically, we found that both naked and PEG-coated AuNPs cause cell death at high concentrations. Quantitative uptake, quantitative cell death rate and colloid concentration appear all correlated.

Quite interestingly, no particle uptake was found at cell nuclei locations. This indicated that the nuclear membrane selectivity remained unchanged in the presence of nanoparticles.

## Results and discussion

### Cytotoxicity

**Figure 1:**
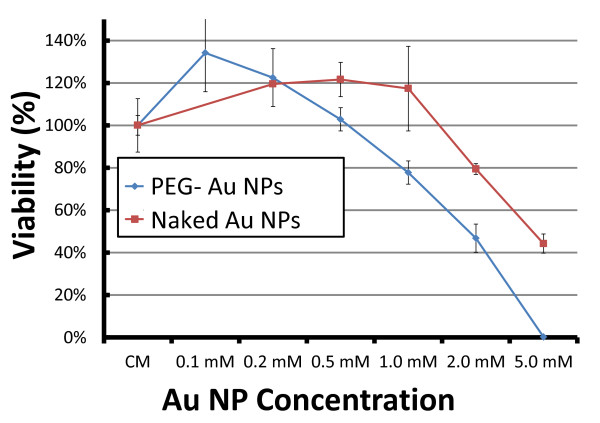
**Results of the cell survival test**. Cell survival test of EMT cells exposed to AuNPs with or without PEG capping. The cells were continuously co-cultured with colloidal nanoparticles for 24 h. The cell viability was measured by direct counting the cell number by trypan blue exclusion. The data are plotted as the percentage of surviving cells compared to untreated control specimens.

The cytotoxicity results for EMT cells exposed to different nanoparticle colloid concentrations and for the control EMT specimen are shown in Figure 1. Cells treated with a 1 mM colloid of bare AuNPs exhibited >95% cell viability. This decreased to 44 ± 4% at 5 mM, indicating that even without surface treatment the AuNPs damage cells, i.e., cellular homeostasis cannot be maintained at high nanoparticle concentrations.

The same figure shows that the PEG coating increased (by 30-40%) the nanoparticle damage at very high concentrations. At low concentrations (0.1 mM), the nanoparticles did not significantly affect the cell viability.

**Figure 2:**
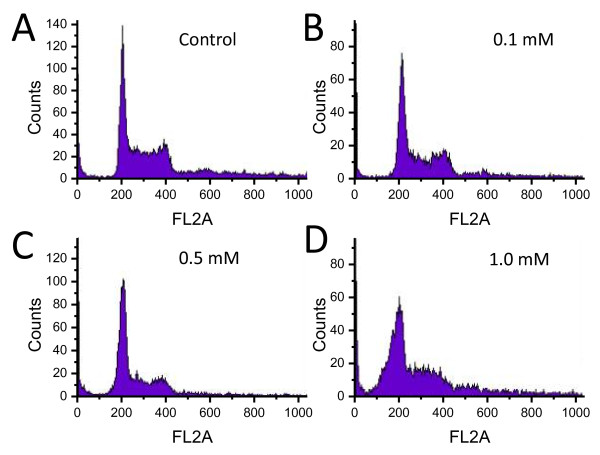
**Results of the flow cytometry**. The flow cytometry profile of the EMT cell cycle after co-culturing with PEG-coated AuNPs with different colloidal concentrations was performed with a fluorescence-activated cell sorter (FACS). There was no significant increase in the apoptotic cells as the nanoparticle concentration increased (A: Control, B: 0.1 mM, C: 0.5 mM, D: 1.0 mM), indicating that the apoptosis is not likely to cause the observed cell damage in this case.

To determine if apoptosis was the cause of cell death for highly concentrated PEG-coated AuNPs, we performed flow cytometry with a fluorescence-activated cell sorter (FACS) [41]. As shown in Figure 2, there was no significant increase in the apoptotic cells as the PEG-coated AuNP concentration increased: the profile is similar to that of the control specimen. This indicates that cell death does not occur via apoptosis but via necrosis.

### TEM imaging

**Figure 3:**
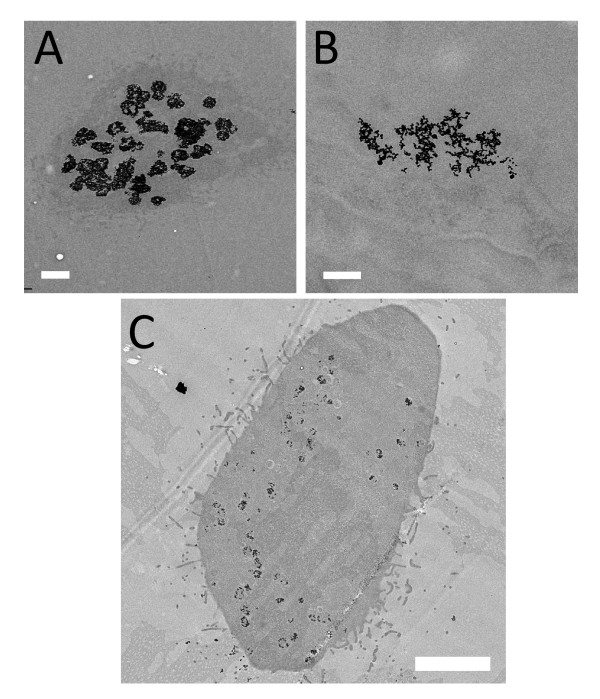
**TEM images of cells with internalized AuNPs**. After co-culturing with 500 μM colloidal naked AuNPs for 48 h, the endocytotic vesicles of these cells were found to contain clusters of many AuNPs inside the cytoplasm. Note that the size of the clusters is significantly larger for EMT cells (A and B) than for HeLa cells (C). Bars: 1 μm (A), 200 nm (B) and 5 μm (C).

TEM was first performed on thin sections, of thickness <100 nm, of all the cell lines with and without exposure to nanoparticle colloid. Figure 3 shows examples of TEM micrographs of the EMT and HeLa cells with AuNPs internalized. After co-culturing with 500 μM naked AuNPs for 48 h, the endocytotic vesicles inside the cytoplasm of cell lines contained clusters of many nanoparticles. However, there are visible differences between the two lines: the vesicle size for EMT cells (A) is substantially larger than for HeLa cells (B).

This is an example of the quantitative information yielded by TEM: the average size of the vesicles in Figure 3A, 3B and 3C are 637 ± 41 nm 530 ± 16 nm and 280 ± 30 nm (n = 5 for EMT cell; n = 4 for HeLa cell). There are, however, limitations in the quantitative data that can be extracted with TEM. The images of Figure 3 are from very thin slices of cells hundreds time thicker, and essentially yield 2D information.

**Figure 4:**
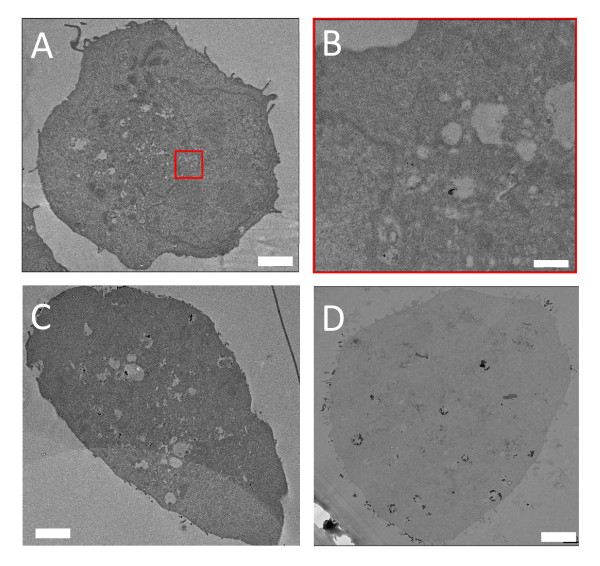
**TEM images of EMT cells for different naked AuNP co-culture times**. A) co-culturing with 500 μM AuNP colloid for 30 min: only a few AuNPs can be found on the surface or inside the cytoplasm. The red square (B) marks the area where clusters are found. C) 1 h co-culture time: a larger number of endocytotic vesicles containing AuNPs is found in the cytoplasm. D) 6 h co-culture time: the number and the size of endocytotic vesicles containing AuNPs are even larger. Bar: 2 μm (A, C and D) and 200 nm (B).

Three-dimensional information can be obtained with TEM by continuous sectioning, but both the image taking and the image analysis are time consuming. This is particularly true if the density of uptaken nanoparticles is low, since the procedure would require the analysis of many slices to obtain reliable results. For example, in Figure 4, TEM images are used to analyze the uptake of naked AuNPs in EMT cells for different co-culture times. After 30 min co-culture, only a very few AuNPs are uptaken and most TEM images show no AuNPs at all. Only by analyzing many such images we found AuNPs on the cell surface or inside the cytoplasm shown in Figure 4A and 4B. This is due to the necessary time for cells to produce and internalize the vesicles packing nanoparticles for endocytosis. This means that for short co-culture times it is difficult and time-consuming to go beyond a mere qualitative analysis.

### X-ray imaging

**Figure 5:**
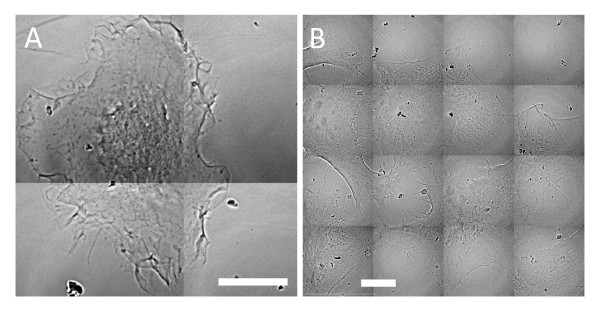
**Transmission x-ray microscopy image of EMT and HeLa cells without chemical staining**. A) EMT cell: some filopodia on the cell boundary are visible. B) HeLa cell: the membrane ruffles and the nuclei are clear. Bars: 10 μm).

**Figure 6:**
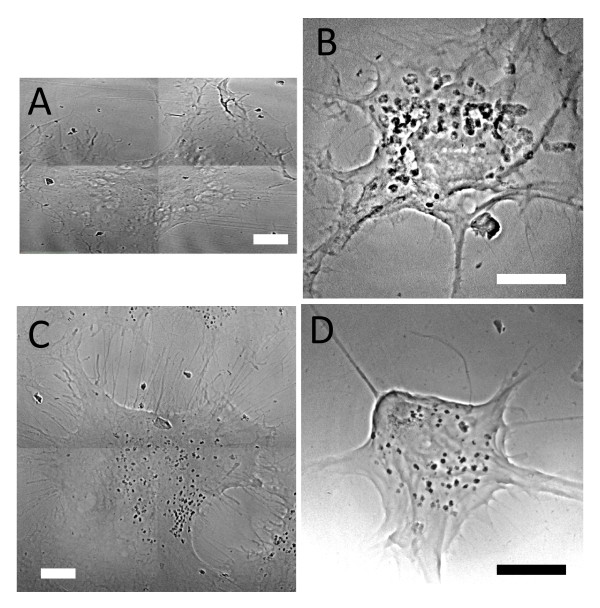
**Transmission x-ray microimages images showing the different internalization of AuNPs by different cell lines**. A) An EMT cell co-cultured with 1 mM PEG‐coated AuNPs for 48 h. B) An EMT cell co-cultured with 500 μM naked AuNPs for 48 h. C) A HeLa cell co-cultured with 500 μM naked AuNPs for 48 h. D) A CT-26 cell co-cultured with 500 μM naked AuNPs for 48 h. (Bars: 5 μm).

Figures 5 and 6 show the main features of our x-ray micrographs in view of the quantitative analysis. Specifically, Figure 5 demonstrates that the details of 2D cultured specimens can be seen even without staining. In fact, the nucleus morphology and some subcellular details are clearly visible for EMT and HeLa 2D cell cultures- see Figures 5A and 5B. However, other organelles such as mitochondrias and vacuoles less dense and smaller than the nuclei were not fully imaged and required staining.

Figure 6 shows that even without staining this imaging method can readily explore in detail the dissimilarities in the internalization of different AuNPs by different cell lines. Specifically, the amount of PEG coated AuNPs uptaken by EMT cells is much less than that of naked AuNPs, as seen in Figure 6A and 6B. Similar differences between these two types of AuNPs were found for all the cell lines (data not shown). For naked AuNPs, different cell lines also exhibited different responses in terms of total amount of internalized nanoparticles and nanoparticle cluster morphology. Comparing Figure 6B and 6C, it is clear that naked AuNPs are more numerous in EMT cells, form larger cluster and are distributed more evenly in cytoplasm than in HeLa cells. For comparison, we also show a similar image of naked AuNPs in CT-26 cells (Figure 6D) revealing a situation intermediate between those in EMT and HeLa cells.

**Figure 7:**
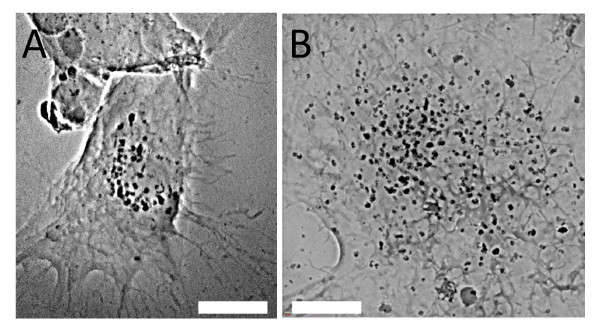
**Transmission X-ray microimages of a 2D cultured HeLa cell**. A) Co-cultured with500 μM naked AuNPs for 48h show the aggregation of AuNP clusters around the cell nucleus (Additional file 1). B) With NAB-Ni staining, AuNP clusters are imaged within the cell skeletons. Bars: 5 μm.

These qualitative conclusions from 2D projection images can be confirmed by examining the specimens from different illumination/imaging directions as shown in Figure 7. The nanoparticle clusters appear at the nuclear membrane location of HeLa cells (Figure 7A and Additional files 1) whereas the much larger clusters inside EMT cells are distributed more uniformly throughout the cell cytoplasm. After specific staining the cell skeleton by the DAB-Ni enhancement method, we found (see Figure 7B) a close relation between the uptaken naked AuNPs and the skeletons. The high lateral resolution enabled us to detect individual naked AuNPs and to conclude from these images that no AuNPs crossed the nuclear membrane.

**Figure 8:**
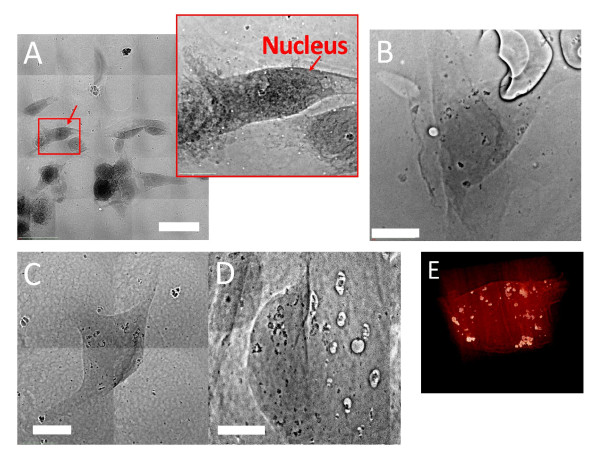
**Transmission x-ray microimages of 3D cultured EMT cells**. EMT cells were grown on an OPLA scaffold. A) Cells from an untreated (without nanoparticles) specimen stained with uranium acetate. The nuclei (one of them marked by an arrow) are clearly visible. Bar: 20 μm. B) EMT cells co-cultured with 500 μM naked AuNPs for 6h. The nucleus would not be visible without staining whereas the AuNPs could be observed for unstained specimens due to their strong contrast. Bar: 5 μm. (C) Patchwork of projection micrograph for an EMT cell co-cultured with 500 μM naked AuNPs. Bar: 5 μm. (D) Single projection images like this were collected for tomographic reconstruction at 1 degree intervals with respect to the incoming x-rays (Additional file 2). Bar: 5 μm. The cell was stained with uranium acetate, targeting the lipid membrane. E) Picture of the 3D tomographic reconstruction of an EMT cell. The nanoparticle cluster distribution can be reliably extracted from the corresponding movie (Additional file 3).

The morphology of 2D cultured specimens could affect the nanoparticle uptake. Therefore, we also performed tests on 3D specimens with tomographic image reconstruction. Figure 8A-C shows an example: Figure 8A is the projection image of a control EMT cell grown on a scaffold, revealing the overall cell shape. The magnified image on the right shows the nucleus (marked by arrow). Figure 8B shows similar results for a cell specimen treated with a nanoparticle colloid. Figure 8C and 8D (movie of different projection in Additional file 2) shows the results of specimen staining in revealing the detailed localization and shape of the nucleus and of the overall nanoparticle cluster distribution. Figure 8E shows the 3D tomographically reconstructed image of an EMT cell after AuNP treatment. The distribution of AuNPs and their specific location in the cell can be obtained from the rendered 3D movies (Additional file 3). It is clear that the AuNPs were not internalized in the cell nucleus. Furthermore, the cluster size distribution was quite similar to the results previously obtained on 2D cultured cell specimens.

**Figure 9:**
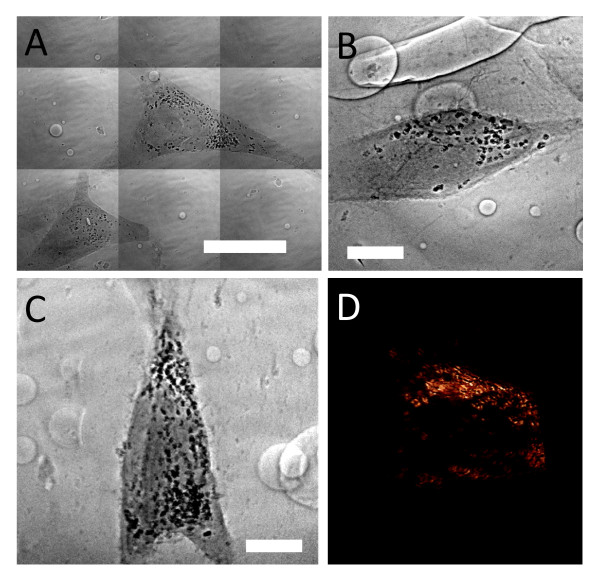
**Transmission x-ray microimages of 3D cultured HeLa cells**. Similar to Figure 7, the HeLa cells were grown on an OPLA scaffold. Bar in A: 20 μm, B and C: 5 μm. (Additional files 4 (9A) and 5 (9B))

**Figure 10:**
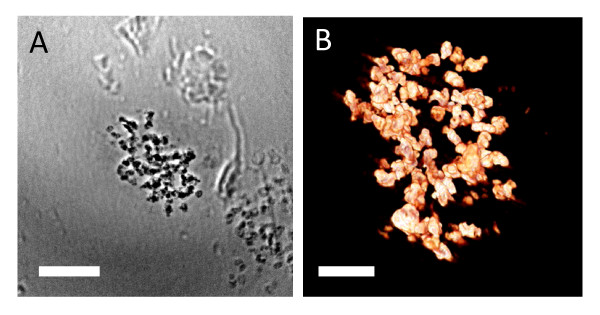
**EMT cells prepared as the pellets**. EMT cell co-cultured with 500 μM naked AuNPs and prepared as the pellets; the specimen was not stained. A) Transmission x-ray projection micrograph (Additional file 6). Bar: 5 μm. B) 3D reconstructed tomography image (Additional file 7). Bar: 5 μm. The distribution of AuNPs is shown in the corresponding movie.

Figure 9 shows results for a HeLa cell 3D culture, treated with AuNP colloid and further stained. Once again, the staining procedure put in evidence the subcellular details and the nanoparticle distribution (Additional files 4 and 5 corresponding to Figure 9C and 9D). Figure 10 (and Additional file 6) shows similar results for a 3D "pellet" EMT specimen. Similar to movies associated with Figure 10B (Additional file 7), the tomographic reconstruction clearly reveals the aggregation of nanoparticles.

### Quantitative data

**Figure 11:**
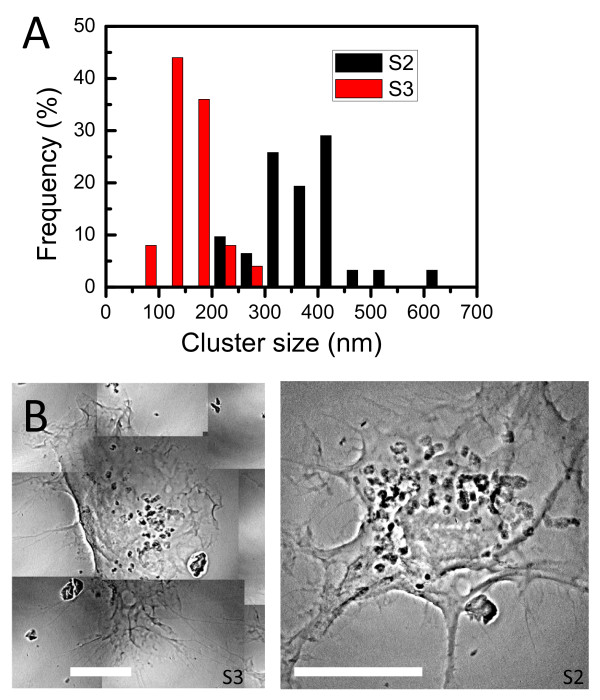
**Quantitative analysis of the uptake of EMT cells by transmission x-ray microimages**. Transmission x-ray uptake microanalysis by EMT cells after co-culturing with 500 μM naked AuNPs for 48 h. The size distribution in A) was obtained by analyzing individual cells such as that in B). Bars in B: 10 μm.

**Figure 12:**
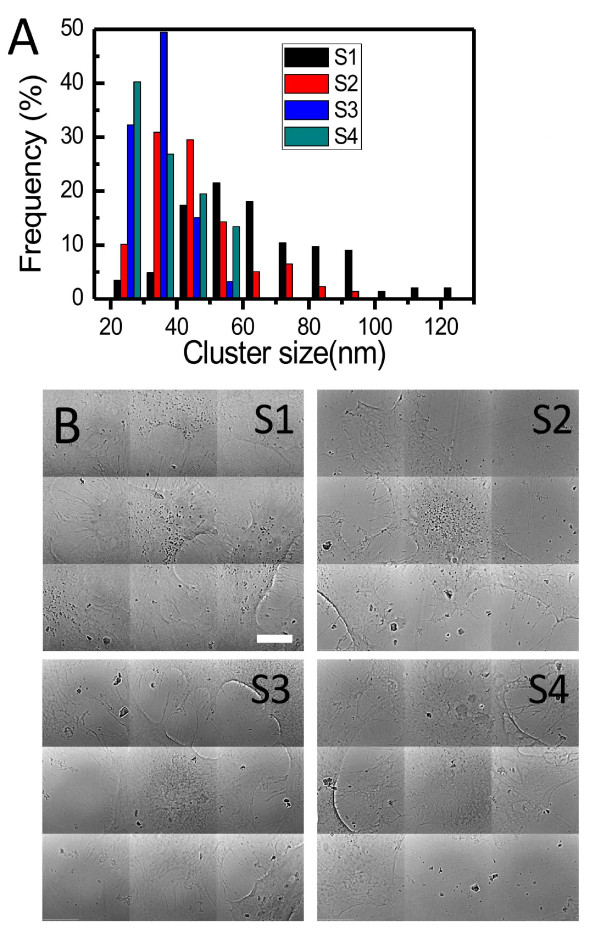
**Quantitative analysis of the uptake of HeLa cells by transmission x-ray microimages**. Results similar to those of Figure 10, for HeLa cells. Bar: 5 μm.

These are the core objective of our present work and the basis for the validation of our method. Figures 11 and 12 show typical results of the procedure: the size distributions of the cluster, formed by aggregation of the internalized AuNPs, for 2D and 3D cultures of EMT and HeLa cell (without staining). The distributions were obtained by analyzing 6 EMT cells and 4 HeLa cells.

These results indicate why the mere evaluation of the AuNP uptake by averaging over a large number of cells is not sufficient to understand the quantitative aspects of the phenomenon. In fact, the above figures reveal substantial differences between different types of cells and even between different cells of the same type.

We see indeed distributions with different peaks and different spreads. Overall, the data for EMT cells suggest a size distribution peak around 140 nm for EMT cells and around 30 nm for HeLa cells. These cluster distribution differences are statistically significant. Thus, our approach made possible a quantitative evaluation of AuNP cluster distributions at the individual cell level, yielding relevant information for the intracellular uptake mechanism that cannot be delivered by cell-averaging procedures.

In addition, our microimages also revealed interesting qualitative differences between EMT and HeLa cells. We see in fact from Figures 11 and 12 that the clusters are concentrated near the nucleus for HeLa cells, whereas they are more uniformly distributed for EMT cells. By measuring the location of the AuNP clusters with respect to the nucleus membrane from 3D images such as Figure 7A, we could determine that the clusters were internalized within a very narrow region, ~1 ± 0.5 μm, outside the HeLa cell nuclear membrane.

The difference of the AuNP internalization process between cell lines can be explained by differences in the biophysical mechanisms: the endocytosis depends on the cell membrane properties, which can be largely different between epithelial, endothelial, cubic, circulating, etc. phenotypes. The different size of the clusters encompassed by endosomes could also result in different intracellular transportation mechanisms of the AuNP clusters. One must also consider that biochemical environment will play a major role in this process, with pH, fluid pressure, and interstitial homeostasis modulating the size and the number of formed vesicles. Further studies on the details of the dynamics of these processes with respect to the size of the clusters are underway.

**Figure 13:**
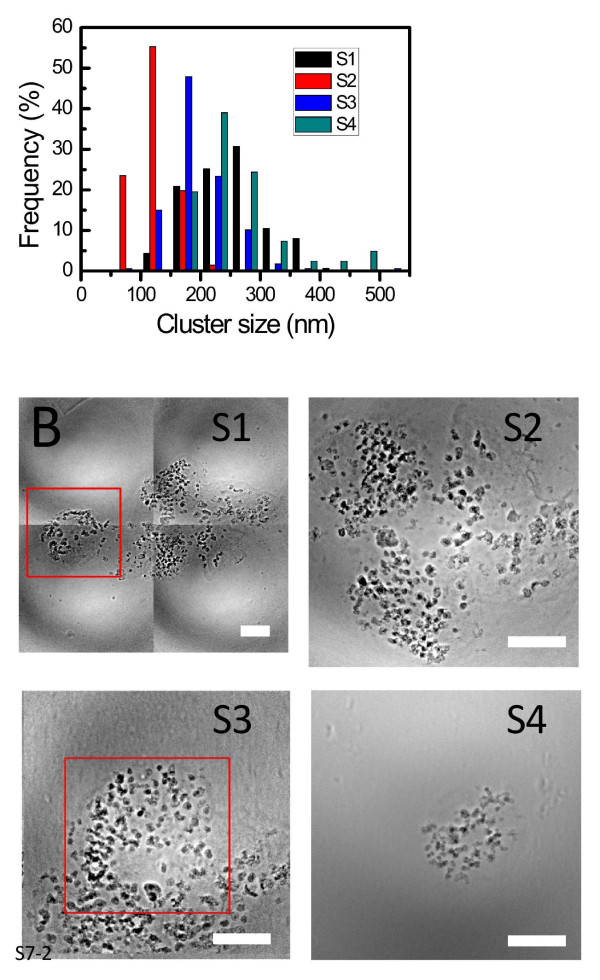
**Quantitative uptake analysis of EMT plellets by 3D images**. A) Internalized cluster size distribution from four x-ray transmission micrographs (B) of 3Dal pellet specimens of EMT cells. Bars: 5 μm. (Additional file 8).

Figure 13 emphasizes another important quantitative issue: the difference between 2D and 3D analysis. The figure shows four x-ray micrographs from 3D pellet specimens of EMT cells. These projection images are from large angular sets (Additional file 8) from which tomographically reconstructed pictures were obtained. The analysis of such pictures yielded the cluster size distribution also shown in Figure 13.

It is clear that such a distribution is substantially different from the corresponding 2D results, Figure 11. This means that the reliable extraction of data on uptaken nanoparticles must include the analysis of 3D specimens, made possible in our approach by the combination of x-ray microscopy and tomographic reconstruction.

On the qualitative side, 3D data on pellet specimens corroborated the information from 2D cultured specimens. Specifically, they confirmed that clusters inside EMT cells are substantially larger and more uniformly distributed than inside HeLa cells, even when the cells are prepared in 3D.

## Conclusions

We experimentally demonstrated that high resolution x-ray micrographs yield important quantitative information about the nanoparticle internalization processes. We specifically found substantial differences in the cluster size distributions and in the overall cluster uptake between individual cells even if they are in the same culture. Similar but quantitatively more important differences were found between different types of cells, together with qualitative differences in the spatial distributions inside the cells. Such results prove not only the feasibility of our quantitative method but its effectiveness and expanded features with respect to other approaches. Substantial differences between 2D and 3D cultured cells as well as between the results of 2D and 3D data analysis stress the importance of 3D procedures like the tomographic reconstruction made possible by our approach.

## Methods

### AuNP synthesis

Bare, MUA and PEG-coated (pegylated) AuNPs in colloidal solution were synthesized by the synchrotron x-ray irradiation method [40,42-44]. A mixture of gold precursor, salt buffer and water was exposed for 5 min to the x-rays emitted by the 01A beamline of the National Synchrotron Radiation Research Center (NSRRC), Hisnchu, Taiwan. The photon energies of this beamline are in the 8-15 keV band. The nanoparticle colloids were then centrifuged using an Amicon ultra-15 centrifugal filer tube (Millipore, Billercia, MA) to increase the concentration and to remove the unreacted precursors.

### Cell culture

EMT-6, CT-26 and HeLa cells were separately cultured in Dulbecco's modified Eagle medium (DMEM)-F-12 medium and DMEM medium (Invitrogen, Carlsbad, CA) supplemented with 1% penicillin-streptomycin and 10% heat-inactived fetal bovine serum (Invitrogen, Carlsbad, CA) and were maintained in a humidified incubator with 5% CO_2 _and at 37 C; the culture medium was changed every two days.

### Cytotoxicitic assay

AuNP colloid was freshly prepared and diluted with Dulbecco's medium. After overnight cell seeding in a multiplate, EMT-6 cells were co-cultured for 24 hours with AuNPs with different colloidal concentrations: 0.1, 0.25, 0.5, 1.0, 2.0, 5.0 and 10.0 mM. Growth medium with no nanoparticles was used for the control specimens. After incubation, some of the cells were harvested and stained by trypan blue reagent (Sigma, St. Louis, MO) to count the number of live cell.

### Flow cytometry analysis of the cell cycle

After a 24-hour incubation, trypsin was used to detach the cells from the petri dishes. The cells were frozen with pre-cooled methanol for 3 min and then stained with propidium iodide for 20 min. Flow cytometry was then performed by FACS Calibur E2594 and the CellQuest Acquisition and Analysis Software (Becton Dickinson Biosciences, Franklin Lakes, NJ) was used to treat the required 5000-7000 cells for each sample.

### Cell preparation for transmission electron microscopy

Cells prepared as described above were deposited on an acryl embedding film. After removing the medium by PBS washing, the sample was fixed with 4% (w/v) paraformaldehyde (EMS, Hatfield, PA) plus 2.5% (w/v) glutaraldehyde (EMS, Hatfield, PA) mixture for 20 min at room temperature. Then, we used a 2% (w/w) osmium tetroxide (EMS, Hatfield, PA) water solution to post-fix it for 30 min. Later, the sample was dehydrated using a series of ethanol solutions with increasing concentration, 30%-100%; each washing step lasted 15 min. Ultrathin sample sectioning, down to ~90 nm, was performed with a diamond knife.

### Cell preparation for transmission x-ray microscopy

Two dimensional cultured specimens were obtained by growing the cells on a Kapton film overnight for complete cell attachment. For 3D specimens, the cells were grown on BD^® ^3D OPLA scaffolds (Becton Dickinson Biosciences, Franklin Lakes, NJ) in vitro. Such scaffolds can be used for a variety of cell types and have a porous architecture suitable for microscopic observations. The fixation was performed using a 4% (w/v) paraformaldehyde and 2.5% (w/v) glutaraldehyde mixture with 1X PBS buffer both for cells grown on Kapton films and on scaffolds. Osmium tetroxide and uranium acetate were used in some cases to enhance the absorption contrast (see the discussion below about absorption vs. phase contrast).

Three-dimensional specimens were also prepared with a "pellet" technique: after the 2D culture described above, the cells were lifted by trypsin. After they developed a spherical form, they were fixed and stained as described above. During the acquisition of projection image sets for tomography, we used Embed-812 Resin (EMS, Hatfield, PA) or photoresist to preserve the specimen structure.

A commercial kit (Vector Laboratories, Burlingame, CA) was used to perform DAB-nickel enhancement staining. The metallic nickel-DAB mixture was imaged exploiting its strong x-ray absorption and used as a contrast agent to image specific subcellular organelles.

### X-ray imaging

The technical details and performances of our transmission x-ray microscope were reported [37,38,45]. The field of view is 24 μm and the detector is a 2048 × 2048 CCD. In the present study, each projection image was collected with a 1-4 s exposure time and then normalized by the background illumination intensity. Images for entire cells were obtained by patchworking several pictures.

For tomography, images were collected at regular angular intervals over 140 degrees (the limits of the experimental geometry). The tomographic reconstruction was performed using an Xradia software and then visualized with the Amira software.

For quantitative analysis, the procedure was interactive. By visual inspection, an appropriate grey scale value was selected to segment each TXM image and identify areas with nanoparticle clusters. The large difference in contrast between cells and nanoparticles makes this segmentation reliable. Then, the number of clusters was interactively extracted for each cell as well as the size of each cluster, thus obtaining cell-specific cluster size distributions.

The particle size analysis in 2D images was performed with image segmentation followed by an approximate fitting of the particles by ellipses. The image segmentation is first performed on tomography reconstructed 2D slice by homemade routine in the Image Pro Plus (Mediacybernetic) software. After integrate the complete set of slices to form 3D model by Amira software overlaid with the segmented objects, the particle size was evaluated as the average of the long and short axes of each ellipse. In 3D, we used ellipsoids rather than ellipses and evaluated the size as the average of the 3 axes in 3 directions. Sampling included only particles that clearly resided inside the cells.

## Competing interests

The authors declare that they have no competing interests.

## Authors' contributions

Conceived and designed the experiments: HSC CCC CP YH. Performed the experiments: HSC CCC CLW IMK. Analyzed the data: HSC CCC EL YH GM. Wrote the paper: HSC YH GM. All authors read and approved the final manuscript.

## Supplementary Material

Additional file 1 **A movie obtained from sequential projection X-ray micrographs taken with 1 degree angle separation of a HeLa cell shows the aggregation of AuNP clusters around the cell nucleus**. Click here for file

Additional file 2 **A movie obtained from sequential projection X-ray micrographs taken of an EMT cell grown on an OPLA scaffold with AuNPs**. Click here for file

Additional file 3 **Movies from pictures of the 3D tomographic reconstruction of an EMT cell with AuNPs**. Click here for file

Additional file 4 **A movie obtained from sequential projection X-ray micrographs of a HeLa cell grown on an OPLA scaffold with AuNPs**. Click here for file

Additional file 5 **Movies from pictures of the 3D tomographic reconstruction of a HeLa cell with AuNPs**. Click here for file

Additional file 6 **A movie obtained from sequential projection X-ray micrographs of a pallet EMT cell with AuNPs**. Click here for file

Additional file 7 **Movies from pictures of the 3D tomographic reconstruction of a pallet EMT cell with AuNPs**. Click here for file

Additional file 8 **A movie obtained from sequential projection X-ray micrographs of specimen S3 (pallet EMT cells) in Figure 13 taken at 1 degree intervals**. Click here for file
